# Kinematic and Neuromuscular Measures of Intensity During Drop Jumps in Female Volleyball Players

**DOI:** 10.3389/fpsyg.2021.724070

**Published:** 2021-09-20

**Authors:** Maximiliano Torres-Banduc, Rodrigo Ramirez-Campillo, David Cristobal Andrade, Julio Calleja-González, Pantelis Theo Nikolaidis, John J. McMahon, Paul Comfort

**Affiliations:** ^1^Escuela de Kinesiología, Facultad de Ciencias de la Salud, Universidad de Las Américas, Viña del Mar, Chile; ^2^Escuela de Ciencias de la Salud, Universidad de Viña del Mar, Viña del Mar, Chile; ^3^Department of Physical Activity Sciences, Universidad de Los Lagos, Osorno, Chile; ^4^Centro de Investigación en Fisiología y Medicina de Altura, Faculty of Health Science, Universidad de Antofagasta, Antofagasta, Chile; ^5^Department of Physical Education and Sport, Faculty of Education and Sport, University of the Basque Country, Vitoria-Gasteiz, Spain; ^6^Exercise Physiology Laboratory, Nikaia, Greece; ^7^Directorate of Psychology and Sport, School of Health and Society, University of Salford, Salford, United Kingdom; ^8^Institute for Sport, Physical Activity and Leisure, Carnegie School of Sport, Leeds Beckett University, Leeds, United Kingdom; ^9^Centre for Exercise and Sports Science Research, Edith Cowan University, Joondalup, WA, Australia

**Keywords:** volleyball, team sport, sports, human physical conditioning, resistance training, plyometric exercise, muscle contraction, electromyography

## Abstract

The aim of this study was to assess drop jump (DJ) performance variables (jump height, contact time, and reactive strength index) concomitant to surface electromyography (sEMG) of lower limb muscles during DJs from different drop heights (intensities). The eccentric and concentric phase sEMG from the gastrocnemius medialis, biceps femoris, and vastus medialis muscles were assessed during all tests, with sEMG activity normalized to maximal voluntary isometric contraction (MVIC). In a cross-sectional, study, 10 amateur female volleyball players (age 22.1 ± 1.8 years; body mass 72.9 ± 15.2 kg; height 1.70 ± 0.08 m) completed DJs from six heights [15–90 cm (DJ15 to DJ90)]. During DJs there was no jump-target box to rebound on to. Results of one-way analysis of variance (ANOVA) showed that the jump height, contact time, and reactive strength index were not significantly (*p* > 0.05) different between drop heights. Mean biceps femoris eccentric and concentric sEMG ranged from 27 to 50%, although without significant differences between drop heights. Mean gastrocnemius medialis eccentric and concentric sEMG remained relatively constant (∼60–80% MVIC) across DJs heights, although eccentric values reached 90–120% MVIC from DJ75 to DJ90. Mean variations of ∼50–100% MVIC for eccentric and ∼50–70% MVIC for concentric sEMG activations were observed in the vastus medialis across DJs heights. The biceps femoris eccentric/concentric sEMG ratio during DJ45 (i.e., 1.0) was lower (*p* = 0.03) compared to the ratio observed after DJ90 (i.e., 3.2). The gastrocnemius medialis and vastus medialis eccentric/concentric sEMG ratio were not significantly different between drop heights. In conclusion, jumping performance and most neuromuscular markers were not sensitive to DJ height (intensity) in amateur female volleyball athletes.

## Introduction

Volleyball is a team sport characterized by periods of short duration (i.e., 3–9 s), high-intensity activities, interspersed with periods (i.e., 10–20 s) of recovery (García-De-Alcaraz et al., 2020). Although the actions performed by players may vary in terms of their individual roles, related to technical and tactical requirements, accelerations, decelerations, jumping, ball-striking, and multidirectional locomotion are common movements (Sheppard et al., 2007). In particular, jumping ability has previously been shown to be related to better performance in volleyball (Ziv and Lidor, 2010). In fact, scoring actions (i.e., spike, block, and serve) are mainly performed while jumping vertically (Sheppard et al., 2007). Indeed, there is a significant increase in the number of jumps per set (113.5–181.3 jumps), from young to elite players in men’s volleyball (De Alcaraz et al., 2017). Accordingly, with the principle of training specificity volleyball players should systematically engage in jump-related training programs to improve sports performance (Gabbett, 2016), in a phased, sequential, manner which also includes the development of maximal force production characteristics (i.e., strength) (Lloyd et al., 2011).

Plyometric exercises are often used in jump-related training programs to improve jumping ability, which play a pivotal role in volleyball performance (Ziv and Lidor, 2010). In fact, volleyball is considered a very “explosive” and fast-paced sport in which plyometric training is widely used (Silva et al., 2019). The drop jump (DJ) is a common plyometric drill, especially among volleyball players (Ziv and Lidor, 2010; Silva et al., 2019) and comprises of a rapid transition between the eccentric-concentric [i.e., stretch-shortening cycle (SSC)] muscle actions (Heiderscheit et al., 1996), allowing greater muscle activation and force *via* stimulation of the muscle spindle (Peng et al., 2011). The alternating eccentric-concentric muscle work also leads to the accumulation of potential elastic energy (*via* the series and parallel elastic components), consequently allowing more work to be performed in the concentric phase (Taube et al., 2012). However, optimal drop height when performing DJs to promote muscle performance is inconclusive (Ramirez-Campillo et al., 2013), especially regarding DJ intensity.

Commonly, athletes perform DJs at increased heights for a greater training intensity stimulus (Peng et al., 2011), as a greater drop height results in an increased duration for acceleration, leading to an increased velocity of the center of mass and therefore an increased momentum on impact with the ground. In this sense, it has been suggested that intensity could be evaluated by examining a variety of kinematic parameters (e.g., jump height) and by assessing the activation of muscle by surface electromyography (sEMG) (Ebben et al., 2008). In practical terms, acute increases in sEMG during training exercises may lead to greater training-related adaptations, as suggested in previous studies with team-sport athletes (Serner et al., 2014). However, the interpretation of this must be done with caution considering there is no simple closed-form or equation that describes this relationship (Disselhorst-Klug and Williams, 2020). The sEMG is commonly used as a marker of intensity in strength training exercise (Krommes et al., 2017), with a quadratic increase in root-mean-square amplitude of the sEMG signal across force levels from 20, 40, 60, 80, and 100% of maximal voluntary contraction (Lenhardt et al., 2009). Moreover, greater intensity usually allow greater training-related adaptations (ACSM, 2009). Regarding DJs, it has been shown that greater drop heights may have a significant effect on sEMG achieved during the rebound jump (Aboodarda et al., 2014), with greater sEMG activity during DJs executed from a 60 cm box (DJ60) than from a 40 cm (DJ40) (Bobbert et al., 1987b) or 20 cm box (DJ20) (Peng et al., 2011), suggesting greater plyometric jump intensity from greater drop heights. Similarly, the use of DJ height that allow athletes to achieve maximal reactive strength index may offer greater training-induced adaptations (Ramirez-Campillo et al., 2018b). In this sense, outcomes such as reactive strength index or sEMG can be used as a proxy for potential adaptations. However, not all studies agree with these assertions (Ebben et al., 2008). Moreover, although power output and reactive strength may augment with initial increases in box drop height, if drop height continues to increase the overall muscle performance may be negatively affected (Flanagan and Comyns, 2008), if the athlete does not possess the appropriate force production capabilities required to rapidly decelerate and accelerate their center of mass. Indeed, such relationships may be modulated by athlete’s characteristics, such as the sex of the athlete and their relative strength capabilities, the latter being particularly relevant among females, including female volleyball players (Beckham et al., 2019; Fuchs et al., 2019).

Although some researchers have assessed kinematic and neuromuscular measures of intensity during SSC actions (Ebben et al., 2008; Ebben et al., 2011), whether the results from such studies are replicable in volleyball players is a matter of further research. Previously, jumping performance and neuromuscular markers (Andrade et al., 2020), as well as biomechanical outcomes (Peng et al., 2019) in male volleyball players, were sensitive to DJ height, although not in a clear dose-response fashion. Regarding female volleyball players, the lack of studies preclude analysis of such phenomenon, although it seems that important differences between male and female volleyball players may be hypothesized (Fuchs et al., 2019). Considering the lack of plyometric training studies conducted among females compared to males (Ramirez-Campillo et al., 2018a) and the increased participation of females in volleyball, particularly at amateur level, more research on this issue is needed. This may help strength and conditioning coaches to prescribe adequate plyometric training loads, as needless extra training (e.g., excessive DJ heights) may expose athletes to greater injury risk, especially among females (Brumitt et al., 2016).

From a biomechanical standpoint as the increased drop height results in increased drop duration and therefore acceleration, this would result in a higher impact velocity, increased momentum, and so a higher braking net impulse (Bobbert et al., 1987a). Due to the aforementioned biomechanical considerations, increased drop heights may impose a suboptimal intensity-related stimulus if the athlete does not possess the appropriate force production capabilities required to rapidly decelerate and accelerate their center of mass. Even among male volleyball players, whom usually have greater strength level compared to females, increased drop height may not be an appropriate intensity-related stimulus (Andrade et al., 2020). Moreover, although reactive strength index and jump height may augment with initial increases in box drop height, further drop height increases are expected to reduce reactive strength index and jump height (Flanagan and Comyns, 2008). Therefore, the main aim of this study was to assess maximal jumping performance and neuromuscular activity in lower limb muscles of amateur female volleyball players after DJ from different drop heights, using markers such as reactive strength index, jump height, contact time, and sEMG. We hypothesized that increasing the drop height during DJ will induce greater contact time among amateur female volleyball players, with greater sEMG, although reduced reactive strength index and jump height.

## Materials and Methods

### Experimental Approach to the Study

This study followed a cross-sectional design. Female volleyball players were assessed for the effects of box height during DJ on sEMG, reactive strength index, jump height, and contact time. Jumps were completed from 15-, 30-, 45-, 60-, 75-, and 90 cm boxes.

### Participants

Ten female amateur volleyball players (age, 22.1 ± 1.8 years; body mass, 72.9 ± 15.2 kg; height, 1.70 ± 0.08 m; body mass index, 25.1 ± 4.2 kg/m2) participated in this study. The participants positions were as follow: middle-blocker (*n* = 2), libero (*n* = 1), spiker (*n* = 5), and opposite-spiker (*n* = 2). Athletes were recruited during the competitive period, where they usually completed one regional-level competition per week. Athletes participated in regular volleyball training sessions for 2 h per day, 3 days per week, during 3 months prior to inclusion in this study. All athletes had ≥2 years of regular training and competition experience in volleyball. At recruitment in this study, most of the volleyball training practice was devoted to technical and tactical drills. Moreover, the participants had no regular experience with resistance training or structured plyometric training in the 3 months preceding this study, particularly with DJs. Inclusion criteria were: (i) healthy by self-report (heart and pulmonary disease, and recent surgeries); (ii) completion of an exhaustive health questionnaire; (iii) without injury history in the past 3 months prior to testing (confirmed by checking their training logs with the team head coach). Exclusion criteria were: (i) any condition considered to affect muscle function (e.g., recent bone fractures) or the measurement protocol (e.g., herniated discs); and (ii) being on medications considered to affect dependent variables (e.g., anabolic steroids). The methods and procedures used were approved by the local university (CODE: N°155/2018-code 195.18) and were based on the latest version of the Helsinki declaration.

### Data Collection

To increase testing reliability and minimize learning effects, participants were familiarized with testing protocols during two 1-h sessions, the week before measurements. During familiarization, participants completed a maximal voluntary isometric contraction (MVIC) test on day one and a series of DJ tests on the next familiarization session, with ≥48 h from previous MVIC testing. During testing sessions, the participants performed the MVIC and (after 5 min of recovery) DJs on the same day (avoiding repositioning of the electrodes). The day before a testing session, participants were instructed to perform a low-intensity workout and maintain their dietary routine. A standardized warm-up was completed before each testing session including 5 min of free running and 5 min of dynamic jumps and dynamic stretching (Andrade et al., 2015). All jump tests were performed on the volleyball court where the participants trained and competed. Participants were asked to use the same sport garments, including the shoes that they usually wore during training and/or competition. The sEMG activity was recorded during each test. All measurements were conducted on the same laboratory, under controlled temperature and humidity conditions. Although we did not control for hormone status nor menstrual cycle phase, all the participants were eumenorrheic and did not take any hormonal contraceptive. Furthermore, previous works did not find differences in the DJ (Sipavičiené et al., 2013) performance over the course of an ovarian menstrual cycle. All tests were conducted and at the same time of day to avoid the influence of circadian rhythms.

#### Anthropometry

Standing height (m) and body mass (kg) were assessed using a stadiometer/mechanical scale (SECA, model 220, Hamburg, Germany) with precisions of 0.1 cm and 0.1 kg, respectively. Subjects were tested in light clothing (shoes were removed). The body mass index was calculated from the two measurements (kg⋅m^–2^).

#### Maximal Voluntary Isometric Contraction

**FIGURE 1:**
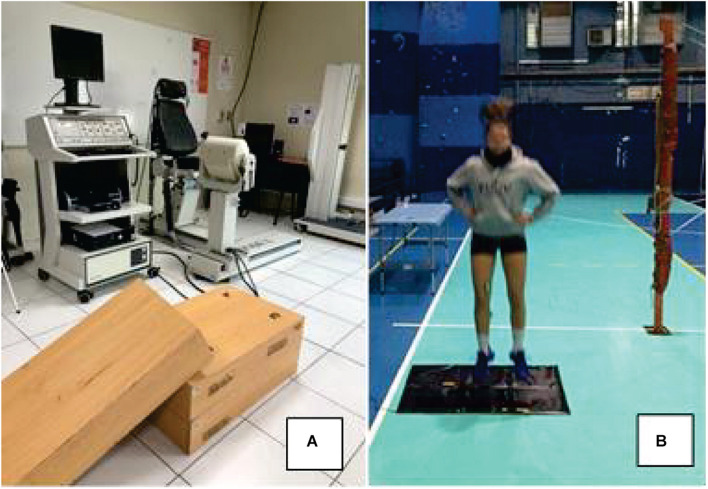
Setup used to evaluate the participants in the study. **(A)** Isokinetic for maximal voluntary isometric contraction and boxes (i.e., 15 cm height each) used during drop jumps and **(B)** contact mat, with participant warming up before measurements.

Following a thorough explanation of the testing procedures, the athletes completed a specific warm-up by completing several submaximal contractions of the knee extensor and plantar flexor musculature. After the warm-up, athletes completed three knee and ankle extension and flexion non-ramped MVIC trials on an isokinetic dynamometer (Biodex^®^, System 3 Pro, NY, United States) (see Figure 1A), with 1 min resting among trials (Ebben et al., 2010). For all MVIC attempts, the gravity effect torque was obtained by weighing the limb with the isokinetic device, and the participants were instructed to perform at maximal effort, given them the instruction to “try to extend your leg (or foot), as hard as you can.” Verbal encouragement was provided during maximal attempts. Each MVIC trial lasted 4–5 s. During the knee extension test, the participants seated in the dynamometer so that their hip and knee joints were positioned at ∼90° angles. Strap was wrapped ∼3 cm above the lateral malleolus of the athlete’s ankle (dominant side), as well as at the thigh, waist, and chest. Care was taken to align the centers of rotation of the knee joint and dynamometer. In the ankle flexion MVIC test, the dynamometer orientation was set at 90°, with a tilt at 0°, seat orientation at 90°, seatback tilt at 70–85°, footplate tilt at 0°, and knee flexion at 20–30°. The axis of rotation was set in neutral position, were axis passes through the body of talus, fibular malleolus, and through or just below the tibial malleolus. The dominant ankle of the athlete (determined by asking the participant the preferred leg used to kick a ball) was attached to the dynamometer with an ankle attachment device, in addition to the use of straps at the chest and waist.

#### Drop Jump Tests

Drop jump heights were randomly selected from 15-, 30-, 45-, 60-, 75-, and 90 cm boxes (DJ15, DJ30, DJ45, DJ60, DJ75, and DJ90, respectively) (see Figure 1A), and participants completed 3 trials at each randomly selected height. An electronic contact mat (Axon Jump 4.0, Bioengineering Sports, Argentina) (see Figure 1B) was used to measure contact time (ms) and flight time, the latter of which was used to calculate jump height. Besides, the reactive strength index was calculated as jump height/contact time (mm⋅ms^–1^), as previously described (Flanagan and Comyns, 2008). Players jumped with arms akimbo and stepped of the box with the leading leg straight to avoid any initial upward propulsion during DJ execution. Upon landing, they were instructed to jump for maximal height and minimal contact time in order to maximize reactive strength index (Barnes et al., 2007). They had 30 s of rest between jumps and 60 s between heights (Andrade et al., 2020). Of note, each athlete completed 18 total jumps (i.e., 3 trials × 6 box heights). Maximal performance (e.g., jump height) was most commonly achieved during the first or second trial (i.e., in 36 of 60 maximal jumps), suggesting lack of learning effect from repeated trials. Additionally, pilot statistical analyses indicated that the mean or the best value (from 3 trials) yielded similar results (i.e., outcomes *p* > 0.05 between DJ heights). Considering that maximal jumping performance may be more ecologically valid for volleyball players, the best performance trial (higher reactive strength index value, associated jump height, and contact time) was used for statistical analysis as previously suggested (Andrade et al., 2020).

#### Surface Electromyography

The sEMG data was acquired (Trigno Wireless System, Delsys, Natick, MA, United States) and used to quantify muscle activity. After warm-up the skin was carefully shaved, abraded and cleansed with alcohol prior to application of EMG + IMU sensor (Trigno Avanti Sensor, Delsys, Natick, MA, United States), whose sample rate of the EMG signal is 1950 and 148 Hz for the accelerometer. As previously suggested (Andrade et al., 2020) the electrodes were placed on the muscle belly surface of the biceps femoris, gastrocnemius medialis and vastus medialis muscles of the dominant leg according to the ‘‘Surface Electromyography for the Non-Invasive Assessment of Muscles (SENIAM)’’ recommendations.^1^ The electrodes were secured with adhesive tape (3M, Canada). The sEMG signals from each electrode were amplified (input impedance 120 kΩ; signal to noise ratio 750; inter-electrode distance of 10 mm) (Ebben et al., 2008) and gain range of 500–5000. Surface electrodes were connected by Wi-Fi to a base station (Trigno Base Station, Trigno Wireless System, Delsys, Natick, MA, United States) and streamed continuously to a computer through an analog to digital converter (G-42, HP notebook computer, United States). The sEMG data was managed with computer software (EMGworks^®^, Delsys, Natick, MA, United States). All data were centered and filtered with a 10 Hz high-pass and a 500 Hz low-pass second order infinite impulse response (IIR) Butterworth filter.

##### Surface electromyography and IMU data recording

The root mean square (RMS) was used to assess sEMG recorded during jump testing (Ebben et al., 2008). Data were calculated using a 60 ms moving window. Data were analyzed to identify the pre- and post-contact muscle burst timing and the magnitude of action for the jump (Ebben et al., 2010). The RMS was evaluated as previously suggested (Gonzalez-Izal et al., 2012):


$$R\,M\,S=\sqrt{\frac{1}{n}\,\sum_{n}x_{n}^{2}}$$

where Xn is the value of the sEMG signal and n is the sample number. During DJ the eccentric and concentric sEMG activity was recorded, coupled with an inertial measurement unit to detect each phase (Trigno Avanti EMG + IMU Sensor, Delsys, Natick, MA, United States). To this aim, the IMU was placed at 1/3 on the line between the tip of the fibula and the tip of the medial malleolus. Behavior analysis of the IMU acceleration signal during a DJ was conducted in a pilot session before the testing sessions. After the application of 10 Hz finite-impulse-response (FIR) filter, signal rectification, and low-pass filtered with a 5 Hz FIR filter, both using a Blackman window (i.e., acceleration impact filter), an initial peak at the beginning of the DJ was noted, corresponding to initial DJ ground contact (start of the eccentric phase), visually determined as the increase in the amplitude of the acceleration (above baseline). Thereafter, the peak progressively descended until return to baseline (i.e., end of the initial peak, end of the eccentric phase, and start of the concentric phase). Another peak was observed after the flight phase of the DJ. Using data from contact time and flight time, calculations allowed the identification of the eccentric and concentric phases. From these recordings, eccentric/concentric ratio was calculated. Each RMS sEMG data was expressed as a percentage of MVIC (Walker et al., 2012) using the highest sEMG recorded during MVIC trials (Andrade et al., 2020). The reliability of these measures has previously been established (Gonzalez-Izal et al., 2012).

### Statistical Analyses

The data used in the statistical analyses is available from the corresponding author upon reasonable request.

The Shapiro–Wilk (*n* < 50) and Levene tests, respectively analyzed the normality and homoscedasticity of the outcome variables. When the data did not confront to the test for normality and/or homogeneity of variance, they were log transformed and were back transformed for presentation purposes.

Data are presented as mean ± SEM. The within-session (between-trial) reliabilities of all dependent variables were assessed by calculating intra-class correlation coefficients and coefficient of variation and the associated 95% confidence intervals. As previously suggested, intra-class correlation coefficients values <0.5, between 0.5 and 0.75, between 0.75 and 0.9, and >0.9 were interpreted as poor, moderate, good, and excellent reliability, respectively, based on the lower bound 95% confidence interval (Koo and Li, 2016).

To calculate the sample size, validated (Faul et al., 2007) statistical software (GPower; University of Dusseldorf, Dusseldorf, Germany) was used. Given the study design (one groups and six repeated measures), a moderate effect size (Cohen’s *f*) = 0.6–1.2 (Andrade et al., 2020), alpha-error < 0.05, the non-sphericity correction € = 1, the correlation between the repeated measures = 0.5, and a desired power (1-β error) = 0.95, the total sample size resulted in six participants. Considering potential dropouts, the minimal initial sample size was set at 10 participants. Considering the characteristics of the sample (i.e., females; amateur level) and the test [i.e., very short duration (<1 s per trial)], a reliability threshold was set at ≤10% for the coefficient of variation (Stokes, 1985).

A series of repeated measures one-way ANOVA tests were used to compare dependent variables collected during the DJ. When a significant *F*-value was achieved, Bonferroni *post hoc* procedures were performed to locate the pairwise differences between the means. Effect sizes (ES) were determined by calculating partial eta-squared ($\eta_{p}^{2}$) derived from ANOVA analysis, converted to Cohen’s *d* values as previously outlined (Fritz et al., 2012) and interpreted using the conventions outlined for sport sciences: <0.2, trivial; 0.2–0.6, small; >0.6–1.2, moderate; >1.2–2.0, large; >2.0–4.0, very large; >4.0, extremely large (Hopkins et al., 2009). Significance was set at an *a priori* alpha level of *p* < 0.05. All statistical calculations were performed using STATISTICA^®^ Software (Version 8.0, StatSoft, Inc., Tulsa, OK, United States).

## Results

For the DJ performance variables contact time, jump height, and reactive strength index, the intra-class coefficients were calculated, for each of the six drop heights, yielding 18 calculations, and 8 demonstrated good reliability, 7 moderate reliability, and 3 poor reliability, based on the lower bound 95% confidence intervals for intra-class correlation coefficients (Table 1). The general trend was for reliability to reduce with an increase in drop height. Regarding the coefficient of variation, 12 out of 18 calculations achieved the ≤10% threshold, with a general trend for reduced reliability in the reactive strength index.

**TABLE 1:** Reliability [intra-class correlation coefficient (ICC) and coefficient of variation (CV)] of drop jump performance variables.^1^

	**DJ15**	**DJ30**	**DJ45**	**DJ60**	**DJ75**	**DJ90**
**Contact time**						
ICC	0.95 (0.82–0.99)	0.96 (0.84–0.99)	0.96 (0.84–0.99)	0.91 (0.69–0.98)	0.97 (0.87–0.99)	0.86 (0.38–0.97)
CV	8.8% (6.0–16.6%)	9.3% (6.3–17.6%)	9.2% (6.3–17.5%)	11.8% (8.0–22.6%)	6.2% (4.1–12.2%)	9.6% (6.1–22.3%)

**Jump height**						
ICC	0.96 (0.84–0.99)	0.92 (0.71–0.98)	0.91 (0.67–0.98)	0.94 (0.76–0.98)	0.87 (0.53–0.97)	0.92 (0.60–0.99)
CV	4.9% (3.3–9.0%)	4.4% (3.4–9.2%)	6.0% (4.1–11.2%)	4.8% (3.2–8.9%)	8.7% (5.8–17.4%)	8.4% (5.3–19.4%)

**Reactive strength index**						
ICC	0.93 (0.76–0.98)	0.93 (0.72–0.98)	0.93 (0.75–0.98)	0.93 (0.73–0.98)	0.82 (0.40–0.96)	0.80 (0.20–0.96)
CV	11.2% (7.6–21.4%)	11.7% (8.6–24.3%)	12.3% (8.3–23.5%)	11.2% (7.6–21.3%)	13.6% (9.0–27.8%)	9.4% (6.0–21.9%)

*^1^Values in parenthesis include 95% confidence intervals.*

The lowest contact time was achieved in the DJ30 (375.2 ± 41.7 ms), and the highest was achieved in the DJ90 (460.6 ± 50.0 ms), with a difference of ∼23%, although no significant (i.e., trivial) differences were observed between drop heights (Table 2). Furthermore, the jump height of the athletes ranged from 27.7 to 29.3 cm, and from 0.66 to 0.87 mm⋅ms^–1^ for reactive strength index, without significant differences between any drop heights (Table 2).

**TABLE 2:** Comparison of drop jump performance variables and surface electromyography (%MVC) between drop heights.

	**DJ15**	**DJ30**	**DJ45**	**DJ60**	**DJ75**	**DJ90**	**ANOVA outcomes Group *F*(5, 50), *p*-value ($\eta_{p}^{2}$)**
Contact time (ms)	384.8 ± 34.2	375.2 ± 41.7	392.8 ± 45.9	377.6 ± 39.5	383.1 ± 34.9	**460.6 ± 50.0**	*F* = 0.5, *p* = 0.78 (0.05)
Jump height (cm)	27.7 ± 1.8	**29.3 ± 1.6**	29.2 ± 1.5	27.8 ± 1.4	29.0 ± 1.5	28.8 ± 1.6	*F* = 0.2, *p* = 0.96 (0.02)
Reactive strength (mm.ms^–1^)	0.76 ± 0.01	**0.87 ± 0.10**	0.84 ± 0.11	0.80 ± 0.08	0.80 ± 0.07	0.66 ± 0.07	*F* = 0.7, *p* = 0.66 (0.06)
BF EC (%MVC)	39.3 ± 9.3	37.0 ± 8.9	37.7 ± 11.0	42.5 ± 11.0	31.6 ± 4.5	**50.0 ± 16.2**	*F* = 0.3, *p* = 0.92 (0.03)
BF CON (%MVC)	28.6 ± 6.8	30.0 ± 7.1	36.0 ± 7.6	**41.8 ± 16.1**	29.5 ± 5.2	27.1 ± 12.6	*F* = 0.3, *p* = 0.90 (0.03)
GM EC (%MVC)	72.7 ± 8.9	62.3 ± 12.5	59.4 ± 8.1	67.8 ± 11.0	93.9 ± 11.7	**117.1 ± 30.3**	*F* = 2.3, *p* = 0.06 (0.19)
GM CON (%MVC)	63.4 ± 9.2	62.9 ± 8.3	75.3 ± 7.0	65.2 ± 5.7	64.7 ± 6.3	**76.3 ± 4.9**	*F* = 0.7, *p* = 0.66 (0.06)
VM EC (%MVC)	68.6 ± 9.0	54.8 ± 6.6	75.2 ± 8.2	95.0 ± 18.2	96.0 ± 11.4	**104.4 ± 24.5**	*F* = 2.1, *p* = 0.08 (0.18)
VM CON (%MVC)	52.5 ± 7.0	65.4 ± 8.9	**72.3 ± 11.8**	52.0 ± 11.0	64.4 ± 9.8	58.1 ± 14.4	*F* = 0.6, *p* = 0.68 (0.06)
BF EC/CON ratio	1.6 ± 0.4	1.4 ± 0.2	1.0 ± 0.1	1.7 ± 0.3	1.5 ± 0.5	**3.2 ± 0.9^*a*^**	***F* = 2.7, *p* = 0.03 (0.21)**
GM EC/CON ratio	1.5 ± 0.4	1.1 ± 0.2	0.9 ± 0.2	1.2 ± 0.3	1.6 ± 0.3	**1.7 ± 0.6**	*F* = 1.1, *p* = 0.38 (0.1)
VM EC/CON ratio	1.4 ± 0.2	1.0 ± 0.2	1.2 ± 0.2	2.4 ± 0.7	1.8 ± 0.4	**2.5 ± 0.9**	*F* = 1.8, *p* = 0.13 (0.16)

*Data are mean ±SEM.*

*BF, biceps femoris; CON, concentric; DJ15-DJ90, drop jump from 15 to 90-cm boxes, respectively; GM, gastrocnemius medialis; EC, eccentric; MVC, maximal voluntary contraction; VM, vastus medialis.*

*^*a*^Significantly greater compared to DJ45 (*p* < 0.05). $\eta_{p}^{2}$: partial eta-squared. Bold values: highest performance and sEMG values for each variable across drop heights.*

The sEMG of the biceps femoris during the eccentric phase varied from 32% (DJ75) to 50% (DJ90), although without significant differences between any drop heights (Table 2). Similarly, the biceps femoris concentric sEMG activation varied between 27 and 42%, without significant differences between any drop heights (Table 2). Similarly, gastrocnemius medialis eccentric sEMG activation remained relatively constant between DJ15 and DJ60 (∼60–70%), with an increase up to 117% in DJ90, although these were not significant between drop heights (Table 2). For the gastrocnemius medialis concentric sEMG, no significant differences were observed between any drop heights, with activation varying between 63 and 76% (Table 2). The vastus medialis eccentric sEMG, although initially showed a reduction from DJ15 (∼69%) to DJ30 (∼55%), thereafter a relatively constant increase was noted up to a maximum of 104% achieved after DJ90 (Table 2), although these were not significant (Table 2). The vastus medialis concentric sEMG highlighted no significant variation (52–72%) between drop heights (Table 2).

**FIGURE 2:**
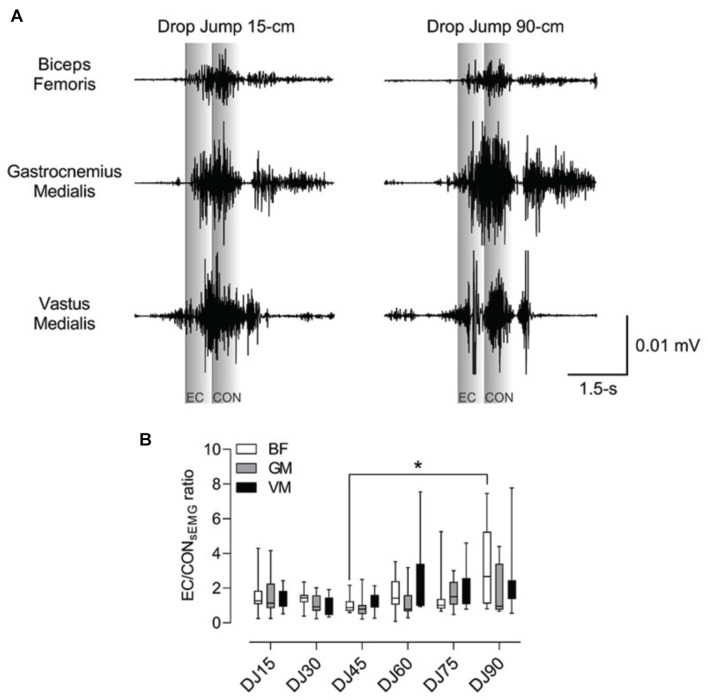
Eccentric (EC) to concentric (CON) surface electromyography (sEMG) ratio in the biceps femoris (BF), gastrocnemius medialis (GM), and vastus medialis (VM) muscles of female volleyball athletes. **(A)** Representative sEMG recording from BF, GM, and VM of one athlete performing drop jumps from 15 cm (DJ15) and to 90 cm (DJ90). **(B)** Note that compared to the EC/CON sEMG BF ratio achieved after DJ45 (i.e., 1.0), a significantly (*p* = 0.03) greater ratio was observed after DJ90 (i.e., 3.2). Data are showed as median and interquartile range, *n* = 10. *Significantly (*p* = 0.03) greater after DJ90 compared to DJ45.

The biceps femoris eccentric to concentric sEMG ratio was relatively constant between DJ15 to DJ75, however, compared to the ratio achieved after DJ45 (i.e., 1.0), a significant (*p* = 0.03; $\eta_{p}^{2}=0.21$; *d* = 1.0, moderate) greater ratio was observed after DJ90 (i.e., 3.2) (Figures 2A,B). The gastrocnemius medialis eccentric to concentric sEMG ratio showed a reduction from DJ15 (i.e., 1.5) to DJ45 (i.e., 0.9), and thereafter a relatively constant increase up to DJ90 (i.e., 1.7), although without significant differences between drop heights (Table 2). The vastus medialis eccentric to concentric sEMG ratio showed variations between 1.0 (DJ30) and 2.5 (DJ90), although without significant differences between drop heights (Table 2).

## Discussion

The main aim of this study was to assess jumping performance and neuromuscular activity in lower limb muscles of female volleyball athletes after DJ from different drop heights, using parameters such as reactive strength index, jump height, contact time, and sEMG. Based on a previous study (Andrade et al., 2020), we hypothesized that increasing the drop height during DJ will not ensure greater kinematic and neuromuscular measures of intensity during plyometric jumps in female volleyball players. Indeed, aside from a greater biceps femoris eccentric/concentric sEMG ratio after DJ90 compared to DJ45, no significant differences were noted between drop heights for the outcomes analyzed in female volleyball players. Therefore, jumping performance and neuromuscular markers were not sensitive to DJ height within these amateur female volleyball athletes.

No significant differences in contact time were noted between DJ15 to DJ90 box heights, whereas a ∼23% increase (although no significant) in contact time was noted in the DJ90 compared to previous drop heights. Relatedly, the reactive strength index, although did not varied significantly across the different drop heights, the lowest value (i.e., 0.7) was achieved after DJ90, a ∼22% reduction compared to the highest reactive strength index value achieved in the DJ30 (i.e., 0.9). Such reduction almost perfectly mimics the increase in the contact time observed in the DJ90. Athletes commonly perform DJs at increased heights for a greater training-intensity stimulus (Peng et al., 2011), with some studies showing greater sEMG activity after DJs executed from a 60 cm box (DJ60) than from a 40 cm (DJ40; i.e., males) (Bobbert et al., 1987b) or 20 cm box (DJ20; i.e., males and females) (Peng et al., 2011). Although this may suggest greater plyometric jump intensity from greater drop heights, our results, in contrast suggest that neither greater sEMG occurred nor greater kinematic (i.e., jump height, reactive strength index, and contact time) performance. Differences between the results of previous studies and our results are not easy to explain. However, participant’s sex seems not a key factor, as previous studies in male volleyball players also found that increasing the drop height during DJs does not ensure a greater training intensity (Andrade et al., 2020). Moreover, similar to our findings, some studies also have noted that greater DJ height may, in fact, reduce performance (i.e., reactive strength index and jump height), for both trained and untrained participants (Flanagan and Comyns, 2008). Therefore, using greater drop height may not always be an efficient strategy to increase training intensity for female volleyball players, as an important reduction in reactive strength index development may be induced, and a suboptimal reactive strength index during training may limit maximization of training-induced adaptations (Ramirez-Campillo et al., 2018b). Moreover, a greater drop height box could increase vertical ground reaction forces (vGRF) (Jensen and Ebben, 2007; Ebben et al., 2008, 2011). Even a DJ30 may induce vGRF ≥4 times body mass in females athletes (McNair and Prapavessis, 1999), potentially contributing to knee instability and non-contact ACL injury (Yu and Garrett, 2007). Therefore, for amateur female volleyball athletes, in order to increase performance and reduce injury risk, the use of moderate height boxes (e.g., ≤DJ30) may be preferred over greater box heights.

Of note, although no significant concentric or eccentric sEMG differences were shown between DJ box heights, the highest values of sEMG activity usually occurred from greater DJ box heights, although the jumping parameters (i.e., reactive strength index) achieved relatively better performance at lower DJs box heights. Reduced jumping performance from higher DJ box drop heights might be related to neuromuscular inhibition, which serves as a protective mechanism to prevent muscle and tendon injury from excessive stress in the muscle-tendon unit (Komi, 2008). However, a greater jumping performance may not necessarily coincide with RMS of sEMG activity (Andrade et al., 2020). This might be explained by the reutilization of elastic energy due to the interaction between contractile elements and a series of elastic elements (Bobbert et al., 1996), gearing greater jumping performance independent of electrical activity. This notion is reinforced by the fact that at greater DJ box height (i.e., 90 cm), a ∼23% increase in contact time was noted compared to lower drop height (i.e., 15 cm), thus increasing elastic energy loss due to a prolonged SSC (Asmussen and Bonde-Petersen, 1974), yielding a relatively reduced jumping performance and an increased sEMG activity. In the present study, sEMG activity was normalized to MVIC to compensate for differences in strength, muscle tone, fat mass, and muscle geometry among other factors that may induce bias in the results (Ankrum, 2000). However, in the literature, different methodologies have been used to assess neuromuscular parameters (Ebben et al., 2008, 2010; Peng et al., 2011), yielding different results. Although no consensus exists (Ebben et al., 2008; Peng et al., 2011), current results agree with the notion that moderate drop heights during DJ allow maximization of performance, including volleyball players (Andrade et al., 2020). In addition, it must be considered that the sEMG eccentric/concentric ratio may be particularly sensitive to changes in jumping intensity (Andrade et al., 2020) as indicated by the greater biceps femoris sEMG eccentric/concentric ratio after DJ90 compared to DJ45. Such result was in line with a tendency for decreased biceps femoris concentric sEMG and increased biceps femoris eccentric sEMG at greater DJ heights. Such findings may be related to the eccentric involvement of the biceps femoris at the hip joint to decelerate the center of mass at landing, with potential implications for hamstring strain injury risk reduction (Bourne et al., 2018).

Taken together, our results suggest that motor unit recruitment does not change across DJ box heights during the eccentric and concentric phases. This observation remains for different muscles (biceps femoris, vastus medialis, and gastrocnemius medialis) and different muscle actions (agonist and antagonist). Therefore, it is debatable if a greater DJ drop height is indicative of greater intensity. Based on recent results, the 50–100% of maximal countermovement jump height may be the appropriate individual relative drop height for the DJ among volleyball players (Peng et al., 2019). Future studies should analyze the effects of adequate (individualized) prescription of drop heights for the DJ on the physical fitness and injury risk among female volleyball players, as previously resolved in other groups of athletes (Ramirez-Campillo et al., 2018a, 2019). Further, DJ drills are meant to be performed usually with a contact time of <250 ms. Of note, although all athletes have ≥2 years of regular training and competition experience in volleyball, is striking that almost all of them did not attain a contact time <250 ms during the different DJ heights. Indeed, only one of the female volleyball athletes managed to achieve such a performance, potentially highlighting that athletes were not appropriately conditioned to gain the maximal benefits from this task. Therefore, relative strength on DJ performance, particularly among female, including female volleyball athletes (Beckham et al., 2019), may be a factor for strength and conditioning coaches and researchers to consider.

### Limitations

Some limitations should be acknowledged. Firstly, it would be risky to extrapolate current findings to other highly trained female volleyball groups, especially those with extensive strength and conditioning preparation, particularly resistance training preparation. Secondly, during the DJ testing session, all the players (except one) achieved contact time >250 ms. On one side, such contact time are commonly observed in the jumping actions of volleyball competitions, meaning high ecological validity for the current findings. However, if current findings may be extrapolated to DJ drills involving fast SSC muscle action, or ground foot contact time <250 ms, also referred as bounce DJ (Bobbert, 1990), should be clarified in future research. Thirdly, drop height is influenced by box height (Geraldo et al., 2019), and a modulation of drop height from greater box heights (e.g., stepping down from the box) may have (inadvertently) occurred, decreasing the height the center of mass drops from. We recommend controlling the center of mass displacement adding cinematic analysis of this variable to confirm or discard the aforementioned. Fourthly, our small sample size may raise some questions on the external validity of these results, particularly since recruited athletes comes from different playing positions (e.g., libero, and setter), and with different training backgrounds (i.e., ≥2 years of volleyball experience). Future studies may consider the inclusion of amateur players but with more years of experience. Finally, the study design (cross-sectional) does not allow inferring cause-effect relationships. Therefore, extrapolation of current findings to training interventions should be performed with caution.

## Conclusion

Our results show that contact time, jump height, reactive strength index, as well as biceps femoris, medial gastrocnemius, and vastus medialis muscles sEMG (i.e., both concentric, eccentric, and eccentric/concentric ratio) were not significantly affected by DJs performed from different drop heights (i.e., DJ15, DJ30, DJ45, DJ60 DJ75, and DJ90), except for a greater biceps femoris eccentric/concentric ratio from DJ90 compared to DJ45. Therefore, DJ performance variables (jump height, contact time, and reactive strength index) concomitant to sEMG of lower limb muscles during DJs from different box heights (intensities) are not sensitive markers to DJ box height (intensity) in amateur female volleyball athletes. In other words, increasing the box heights for DJs may not induce greater training stimulus (i.e., intensity).

## Data Availability Statement

The original contributions presented in the study are included in the article/supplementary material, further inquiries can be directed to the corresponding author/s.

## Ethics Statement

The studies involving human participants were reviewed and approved by the local university (UST, CODE: N155/2018-code 195.18) and were based on the latest version of the Helsinki declaration. The patients/participants provided their written informed consent to participate in this study.

## Author Contributions

All authors made significant contributions, including preparation of the first draft of the manuscript, data collection, analysis of data, interpretation of data, and provided meaningful revision and feedback. All authors read and approved the final manuscript.

## Conflict of Interest

The authors declare that the research was conducted in the absence of any commercial or financial relationships that could be construed as a potential conflict of interest.

## Publisher’s Note

All claims expressed in this article are solely those of the authors and do not necessarily represent those of their affiliated organizations, or those of the publisher, the editors and the reviewers. Any product that may be evaluated in this article, or claim that may be made by its manufacturer, is not guaranteed or endorsed by the publisher.

## Footnotes

1
http://www.seniam.org
